# Challenges and shifting treatment strategies in the surgical treatment of locally advanced rectal cancer

**DOI:** 10.1002/ags3.12349

**Published:** 2020-06-11

**Authors:** Ho Seung Kim, Nam Kyu Kim

**Affiliations:** ^1^ Division of Colorectal Surgery Department of Surgery Yonsei University College of Medicine Seoul Korea

**Keywords:** early recurrence, locally advanced rectal cancer, neoadjuvant, radiation, total neoadjuvant treatment

## Abstract

The current standard treatment for locally advanced rectal cancer (LARC) in Korea and Western countries is a multimodal approach incorporating preoperative long‐course chemoradiotherapy (LCRT) followed by total mesorectal excision (TME) and adjuvant chemotherapy. This approach has significantly improved local control and reduced recurrence rates; however, the overall survival benefit has not been established. Although LCRT is a good option, there remain challenging unresolved problems for colorectal surgeons. We focused on four challenging issues in this review article. The first is LARC with resectable liver metastases, for which there has been no consensus regarding optimal management and practice thus far. The second is cancer progression at the time of restaging after completion of preoperative LCRT. To date, there have been few reports on this issue. The third is early recurrence after TME following preoperative LCRT, the reason for which is thought to be the delayed systemic chemotherapy in the preoperative LCRT protocol. The fourth is cost‐effectiveness. The preoperative LCRT protocol takes 5 weeks. After a 6‐8‐week waiting period, surgery is performed. Therefore, it is more time‐consuming than short‐course chemoradiotherapy. To overcome these issues, total neoadjuvant treatment (TNT) modalities, performed using various protocols, have been conducted globally based on cumulative experience. We also attempted to discuss previous TNT protocols in this article. One treatment strategy is not sufficient for patients with varying clinical characteristics. Therefore, we should revisit current treatment strategies based on recent clinical experience.

## Graphical abstract

One treatment strategy is not sufficient for patients with locally advanced rectal cancer. We should revisit current treatment strategies based on recent clinical experience.

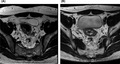

## 1 CURRENT TREATMENT STRATEGIES FOR LOCALLY ADVANCED RECTAL CANCER

Before discussing the treatment of locally advanced rectal cancer (LARC), we should define the rectum. In Japan, the rectum is divided into the rectosigmoid (the sacral promontory to the inferior border of the second sacral vertebra), upper rectum (the second sacral vertebra to the peritoneal reflection), and lower rectum (the peritoneal reflection to the superior border of the puborectal sling).^1^ However, the definition of the rectum is different in Japan from those in Western countries and Korea. Moreover, the definition differs even among Western countries. In the national comprehensive cancer network (NCCN) guidelines, the rectum is defined as the portion of the bowel located below the pelvic inlet (an imaginary line drawn from the sacral promontory to the top of the pubic symphysis) as determined by dedicated magnetic resonance imaging (MRI) of the pelvis. The rectum is divided into the upper rectum (above the anterior peritoneal reflection), mid‐rectum (at the anterior peritoneal reflection), and lower rectum (below the anterior peritoneal reflection).^2^ In the European Society for Medical Oncology (ESMO) guideline, tumors with 15‐cm distal extension from the anal margin (as measured by rigid sigmoidoscopy) are classified as rectal cancers. Cancers are categorized as low (up to 5 cm), middle (>5‐10 cm), or high (>10 up to 15 cm).^3^ In Korea, we use a definition similar to that in the ESMO guideline, in which the rectum is divided into the upper, mid, and lower rectum by 4‐5 cm. In this review article, we used the definition from the Western guideline, as most of the introduced studies and treatment strategies were from Western countries.

Traditionally, preoperative long‐course chemoradiotherapy (LCRT) combined with radiosensitizing chemotherapy followed by total mesorectal excision (TME) and postoperative adjuvant chemotherapy has been used for LARC in Western countries and Korea. In the German CAO/ARO/AIO‐94 trial, neoadjuvant treatment, consisting of 50.4‐Gy radiation in 28 fractions, with concurrent chemotherapy and continuous 5‐fluorouracil (FU) infusion, resulted in significantly better outcomes than adjuvant treatment in terms of lower local recurrence (LR) (6% vs 13%, *P* = .006) and lower toxicity rate (27% vs 40%, *P* = .001).^4^ Neoadjuvant CRT is better tolerated than postoperative CRT, allows curative resection after downstaging, and results in improved sphincter preservation rates. The German trial established preoperative LCRT as the preferred concurrent option for LARC.

Several European studies have looked at the efficacy of preoperative short‐course radiotherapy (SCRT) (25 Gy over 5 days). The results of the Swedish Rectal Cancer Trial, evaluating the use of SCRT administered preoperatively for resectable rectal cancer, showed a survival advantage and a decreased rate of LR compared with the use of surgery alone.^5^ The Dutch TME trial on SCRT reported that 10‐year survival was significantly improved in patients with stage III disease and a negative circumference resection margin (CRM) in the RT plus surgery group compared to the surgery‐only groups (50% vs 40%, *P* = .032).^6^

Along with RT, adjuvant chemotherapy is a matter of debate. Adjuvant chemotherapy is currently recommended in patients with rectal cancer who have undergone upfront surgery and are in postoperative pathologic stages II or III.^7^ Nevertheless, adjuvant chemotherapy remains controversial, especially in patients who undergo preoperative CRT, with no study to date having clearly delineated the role of adjuvant chemotherapy in this setting. To date, adjuvant 5‐FU has been demonstrated to improve survival; oxaliplatin‐based adjuvant chemotherapy has recently been shown to improve disease‐free survival (DFS) in patients with rectal cancer with ypStage II and III disease after preoperative LCRT.^8^

Currently, total neoadjuvant therapy (TNT) has been highlighted as the treatment modality for LARC. TNT is defined as chemotherapy using cycles of induction or consolidation in conjunction with standard CRT prior to surgery. The rationale for TNT includes early introduction of systemic treatment to address micrometastases, earlier reversal of diverting ileostomy, decreased toxicity rates, and increased tumor regression that can enhance complete (R0) resection rates and optimize adaptive strategies and patient selection for organ preservation.^9^ In a recent systemic review and meta‐analysis, TNT increased the risk of pathologic complete response (pCR) by 39% (*P* = .01). Moreover, patients who received TNT and surgery had a better DFS (Hazard ratio [HR] = 0.75, 95% confidence interval [CI] 0.52‐1.07, *P* = .1) and OS (overall survival) (HR = 0.73, 95% CI 0.59‐0.9, *P* = .004) than those who received standard CRT.^10^

In this review, based on accumulated clinical experience, we would like to address a couple of issues that have proven very challenging in the treatment of patients with LARC.

## 2 CHALLENGING ISSUES

### 2.1 LARC with resectable liver metastases

In guidelines on colorectal cancer, if both distant metastases and the primary tumor are resectable, curative resection of the primary tumor is performed and resection of the distant metastases is considered.^2^, ^3^, ^11^ The NCCN guideline also recommends upfront chemotherapy as the primary treatment even in resectable synchronous liver metastases (LM).^2^ However, currently, there is no consensus regarding optimal management and practice.

In rectal cancer with synchronous LM, a more complex procedure for the determination of optimal treatment strategies is required, particularly when patients present with LARC. This is because local treatment with RT is definitely needed for tumor regression of the primary tumor. Delay of administration of systemic therapy has become a great concern because of the potential progression of LM beyond resectability during preoperative LCRT. Accordingly, there is a need for early administration of preoperative systemic chemotherapy and shortened duration of RT owing to the metastatic condition.^12^ Chemotherapeutic agents used in LCRT include 5‐FU regimens that are used as radiosensitizers.^13^ The dose is too small for systemic control of LM.

**Figure 1:**
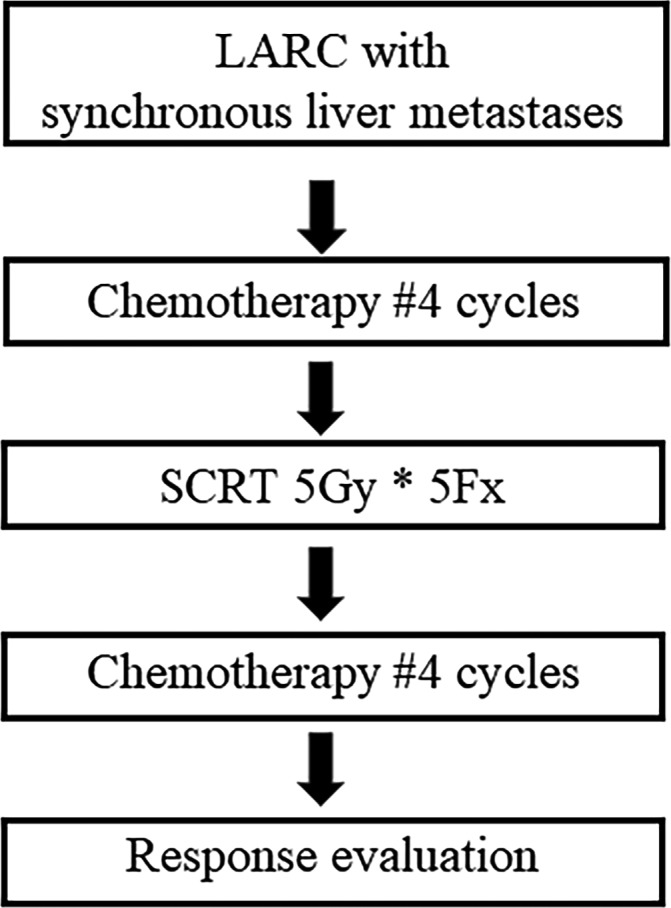
Sandwich protocol

For systemic and local treatment simultaneously, we instituted our own “sandwich” protocol in our institution (Figure 1). This aimed to systemically control first LM; then, SCRT with delayed surgery was adopted for LARC. We administer four cycles of systemic chemotherapy as induction chemotherapy, followed by SCRT for 5 days. Subsequently, additional chemotherapy of the same regimen can be administered during the waiting period to allow tumor regression. After restaging, simultaneous resection for primary rectal cancer and LM was performed. This protocol allows systemic chemotherapy to be administered earlier, and surgery can be planned after the evaluation of tumor response at least 6 weeks after completion of SCRT. A case series of six patients with LARC with synchronous and potentially resectable distant metastases was reported in 2011.^14^ The investigators reported that R0 resection was achieved, except for a primary tumor in one patient. Neither LR nor mortality was observed, and there were acceptable adverse events. This protocol can also be adopted in LARC with LM, even though they are unresectable. A phase II single‐arm study for LARC with synchronous liver‐only metastases was conducted.^15^ This study was focused on LARC with synchronous liver‐only metastases (no number or size limitation of LM). Among 32 patients, surgical resection was performed in 25 patients (78%), and an R0 resection rate of 63% for both sites was recorded. The tumor downstaging rate was 54%. We are still waiting for long‐term outcomes in these patients.

In addition to a primary tumor‐first approach, a liver‐first approach was first described in 2006 in an effort to overcome progression of LM.^16^ The liver‐first approach consists of upfront chemotherapy followed by liver resection and subsequent CRT and primary tumor resection. The theoretical basis is that prognosis of LM may depend more on LM than on the primary lesion. Possible benefits of the liver‐first approach are the early use of systemic chemotherapy and minimization of progression of LM. Andrea et al reported comparable oncological outcomes of the liver‐first and primary‐first approaches (5‐year DFS: 30% vs 29%; 5‐year OS: 42% vs 55%, no statistical difference).^17^ Because there are various treatment modalities, this remains a challenging issue despite the fact that a number of patients do not show LARC with synchronous LM.

### 2.2 Cancer progression at the time of restaging after completion of preoperative LCRT

The second issue is the progression of the cancer at restaging after completion of preoperative LCRT. After 5 weeks of radiotherapy, 6‐8 weeks of waiting period is necessary to evaluate the tumor response. With this restaging time, systemic failure can sometimes be observed. In this situation, depending on the tumor extent, treatment strategies such as surgery for lesions or salvage chemotherapy may be implemented.

To date, there have been few reports on this issue because of the small number of patients. Choi et al reported this issue.^18^ Patients who developed distant metastases within 6 months after CRT were identified. The indications for preoperative LCRT were T4, CRM involved or threatened, or suggestive metastasis of a lateral pelvic lymph node (LN), defined as an LN beyond the TME plane such as iliac and obturator LNs, on initial staging. Among 107 patients who underwent neoadjuvant LCRT, seven (6.5%) developed early systemic recurrence.

Recently, we investigated patients who had distant metastases during the restaging period after preoperative LCRT for LARC (unpublished). Patients with metastases at the initial diagnosis were excluded; 1.3% of patients showed distant metastases during the restaging period. They showed a high incidence of positive mesorectal LN, positive MR‐CRM, and positive MR‐extramural venous invasion (EMVI) during initial clinical staging on magnetic resonance imaging (MRI) (85.7%, 85.7%, and 71.4%, respectively). Poor response in the primary tumor was also observed (mrTRG 3 [MRI‐detected tumor regression grade]: 57.1%, mrTRG4: 35.7%, and mrTRG5: 7.1%).

For these patients, another treatment strategy should be adopted at first. Unfortunately, risk factors for poor response have not been well studied. Further research on this issue is necessary. Proper treatment strategy when patients are expected to be poor responders should be considered. TNT including induction or consolidation chemotherapy can be a possible alternative.

### 2.3 Early recurrence after TME following preoperative LCRT

Early recurrence occurring within one year after surgery for primary rectal cancer has been linked to systemic failure and poor survival.^19^ Early recurrence reflects aggressive biologic behavior. This is the reason early systemic chemotherapy is necessary. Delayed systemic chemotherapy in the preoperative LCRT protocol can be the reason for no benefit on OS, despite the fact that local control may have been obtained.^20^

**Figure 2:**
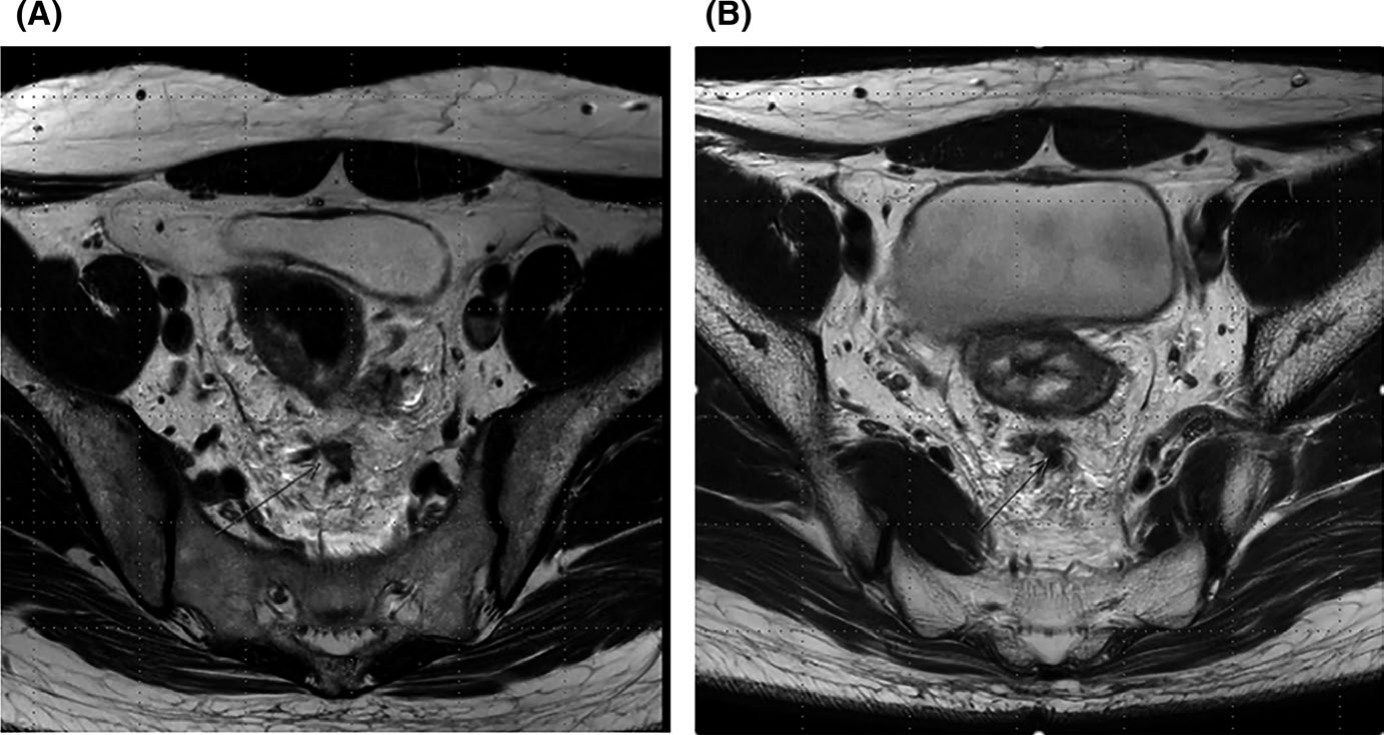
MRI revealing EMVI positivity. A, before neoadjuvant LCRT, B, after neoadjuvant LCRT. EMVI, extramural venous invasion; LCRT, long‐course chemoradiotherapy; MRI, magnetic resonance imaging

Recently, EMVI has been studied in detail (Figure 2). EMVI is defined as tumor cells invading the veins beyond the muscularis propria; it is relatively common in T3‐4 tumors.^21^ EMVI is closely associated with local and systemic recurrence.^22^ Studies have shown the advantages of high‐resolution MRI in detecting EMVI.^23^ When we compared patients with and without mrEMVI after preoperative LCRT, significant differences were observed in terms of 5‐year DFS (80.8% vs 57.8% *P* = .005) and 5‐year systemic recurrence‐free survival rates (86.9% vs 64.3%, *P* = .007). Furthermore, mrEMVI was a significant independent prognostic factor for systemic recurrence (HR: 3.321, 95% CI: 1.185‐9.309, *P* = .022).^24^

For patients who show poor response to LCRT, consolidation chemotherapy consisting of systemic chemotherapy after LCRT before TME can be an option. The current treatment strategy for LARC is preoperative LCRT followed by TME, regardless of tumor regression grade, although poor prognosis can be expected. Marco et al^25^ reported that consolidation chemotherapy increases compliance and DFS in patients with LARC. In terms of SCRT, Bujko et al reported comparable results of SCRT followed by consolidation chemotherapy compared with preoperative LCRT (3‐year DFS: 53% vs 52%, *P* = .85, 3‐year OS 73% vs 65%, *P* = .046).^26^ The ongoing RAPIDO trial (NCT01558921)^27^ will report results soon. The RAPIDO trial compared two groups; the standard arm consists of LCRT preoperatively, followed by selective postoperative adjuvant chemotherapy. Postoperative chemotherapy is optional. The experimental arm consists of SCRT followed by full‐dose chemotherapy before surgery. In the experimental arm, no postoperative chemotherapy is prescribed. The hypothesis is that SCRT with neoadjuvant chemotherapy will increase DFS and OS without compromising local control. The primary end‐point is DFS at 3 years.

When we consider that most early recurrences after preoperative LCRT followed by TME are systemic failures, systemic chemotherapy rather than radiation therapy should be adopted earlier. It is not clear if consolidation chemotherapy can prevent early systemic failure. Further studies of risk factors for early systemic failure and treatment protocols including early administration of systemic chemotherapy are necessary. The standard preoperative LCRT is insufficient in these patients.

### 2.4 Cost‐effectiveness

The preoperative LCRT protocol takes 5 weeks with treatment administered for 5 days per week. After 6 to 8 weeks of the waiting period, surgery is performed. By contrast, SCRT requires only 5 days and a restaging period. There have been many reports comparing outcomes between LCRT and SCRT. However, there have been no reports on cost‐effectiveness among treatment modalities.

Polish and Australian randomized studies have compared preoperative SCRT and immediate surgery with LCRT and delayed surgery in resectable rectal cancer.^26^, ^28^ Ngan et al compared SCRT followed by early surgery and LCRT with delayed surgery.^28^ They found no difference in long‐term outcomes (3‐year LR 7.5% vs 4.4%, *P* = .24; 5‐year distant recurrence 27% vs 30%. *P* = .92; 5‐year OS 74% vs 70% *P* = .62). The Stockholm III randomized study compared preoperative SCRT and immediate surgery with SCRT and delayed surgery.^29^ Surgical complications were more common in SCRT and immediate surgery (36% vs 28% HR: 0.70 CI: 0.51‐0.96, *P* = .03). However, there was no difference in long‐term outcomes. They suggested that SCRT with delayed surgery would be a useful alternative to conventional SCRT with immediate surgery. They also compared the SCRT regimen with LCRT with delay. The cumulative incidence of LR was not different among the groups. They concluded that LCRT with delay is similar to both SCRT regimens; however, it substantially prolongs the treatment time.

**Figure 3:**
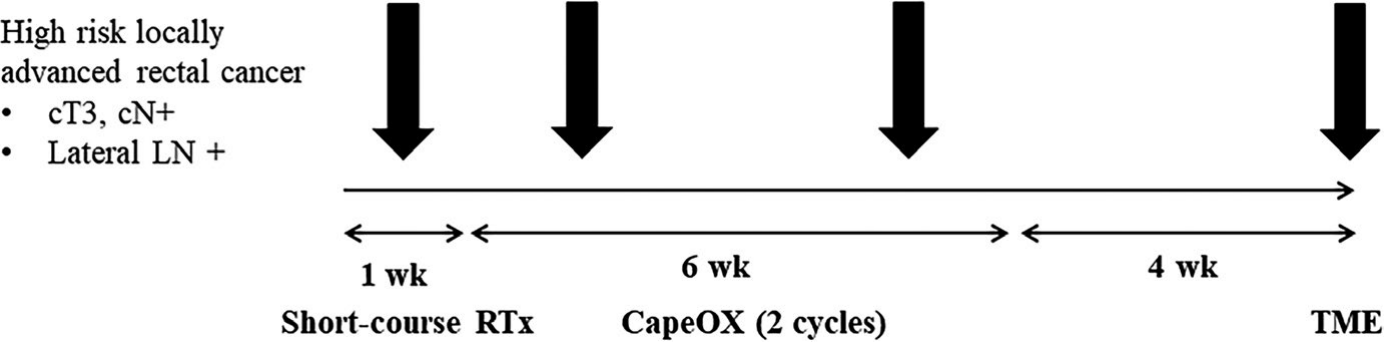
ESCORT (Evaluation of Efficacy, Quality of Life and Cost Effectiveness of Short‐course Radiotherapy Followed by Capecitabine Plus Oxaliplatin chemotherapy and TME for High‐risk Rectal Cancer) protocol

Recently, we started a prospective study called the ESCORT (Evaluation of Efficacy, Quality of Life, and Cost Effectiveness of Short‐course Radiotherapy Followed by Capecitabine Plus Oxaliplatin Chemotherapy and TME for High‐risk Rectal Cancer) (NCT03676517) trial (Figure 3). The main concept of this study is SCRT with delayed surgery. Two cycles of CapeOX are administered during the restaging period. We expect comparable oncologic results to those of previous studies. The strength of our study is the secondary outcome. We have a plan to obtain results for quality of life and cost‐effectiveness. Our hypothesis is that there will be better cost‐effectiveness in patients using the ESCORT protocol with similar oncological outcomes. In circumstances of comparable outcomes between SCRT and LCRT, SCRT can be a good treatment protocol as well as LCRT based on Korean medical environment, in which there is a trend toward patients visiting large hospitals regardless of cost and distance.

Radiation toxicity and effectiveness should also be considered. The PROSPECT trial (NCT01515787) was designed to determine whether neoadjuvant chemotherapy with 5‐FU and oxaliplatin (FOLFOX) could be used as an alternative to neoadjuvant chemoradiation without compromising treatment outcomes to spare these patients excess toxicity.^30^ This trial focused on avoiding unnecessary radiation and toxicity, especially low‐risk LARC patients. Schrag et al^31^ conducted a single‐institution pilot single‐group phase II trial of 32 patients and suggested that it was safe to selectively omit chemoradiation for rectal cancer patients with evidence of clinical response to neoadjuvant FOLFOX chemotherapy. In our study (unpublished), neoadjuvant CRT was related to late anastomosis leakage complications compared with early anastomosis leakage (odds ratio [OR]: 3.032 CI: 1.947‐4.722, *P* < .001). As for the choice between LCRT and SCRT, radiation toxicity should be considered when determining the treatment modality.

## 3 SHIFTING TREATMENT STRATEGY TO TOTAL NEOADJUVANT TREATMENT

Table 1 displays various TNT protocols discussed in this article. Several TNT protocols have been studied worldwide. Some protocols comprising full doses of chemotherapy for 3‐4 months followed by standard CRT and surgery as well as other courses of treatment, including SCRT after chemotherapy (induction chemotherapy), have been studied. Alternatively, some researchers have delivered chemotherapy immediately (consolidation chemotherapy) before surgery and after RT. Another group tried to omit RT according to the response to induction chemotherapy. Target agents were also studied with various treatment strategies.^10^

**Table 1:** Various total neoadjuvant treatment protocols in the review article

Author	Study design	Treatment group	Primary outcome
Marco et al^25^	Nonrandomized	Group I: neoadjuvant LCRT Group II: Neoadjuvant LCRT – Consolidation CTx 2cycles Group III: Neoadjuvant LCRT – Consolidation CTx 4cycles Group IV: Neoadjuvant LCRT – Consolidation CTx 6cycles	pCR rate Surgical difficulty Postoperative complication
Bujko et al^26^	Randomized	neoadjuvant LCRT vs. Conolidation CTx following SCRT	R0 resection
RAPIDO trial ^27^	Randomized	neoadjuvant LCRT vs. Conolidation CTx following SCRT	DFS after 3 years
Fernandez‐Martos et al^32^	Nonrandomized	neoadjuvant LCRT vs. Induction CTx followed by LCRT	pCR rate
Cercek et al^33^	Retrospective	neoadjuvant LCRT vs. Induction CTx followed by LCRT	pCR rate Group I:21%, group II:36%
CAO/ARO/AIO‐12 trial^35^	Randomized	Induction CTx followed by LCRT vs. Conolidation CTx following SCRT	pCR rate
PROSPECT trial^30^	Randomized	neoadjuvant LCRT vs. induction CTx with or without RTx	R0 resection DFS after 3 years

Note

Abbreviations: CTx, chemotherapy; DFS, disease free survival, RTx, radiotherapy; LCRT, long‐course chemoradiation therapy; pCR, pathologic complete response; SCRT, short‐course chemoradiation therapy.

In the Spanish GCR‐3 randomized phase II trial,^32^ patients were randomized to receive CapeOX either before CRT or after surgery (induction chemotherapy group vs adjuvant chemotherapy group). Similar pathologic complete response rates were seen, and induction chemotherapy appeared to be less toxic and better tolerated. A single‐institution retrospective cohort analysis of patients with LARC compared traditional neoadjuvant CRT followed by resection with the TNT approach of induction chemotherapy followed by CRT before resection. Patients in the TNT group received a greater percentage of the planned chemotherapy dose than those in the adjuvant chemotherapy group. The complete response rates were 36% and 21% in the TNT and adjuvant chemotherapy groups, respectively.^33^ Possible benefits of using systemic chemotherapy include the early prevention of eradication of micrometastases, higher rates of pathologic complete response, minimizing the length of time patients need for an ileostomy, facilitating resection, and improving the tolerance and completion rates of chemotherapy. Another benefit can be patient selection for organ preservation. Since the introduction of the “watch‐and‐wait” protocol, non‐operative management has been performed in selected patients with clinical complete response after TNT. An MSKCC‐based multi‐institutional phase II trial (NCT02008656) is being conducted to investigate the efficacies of TNT and selective non‐operative management in LARC. Participants with clinical complete response will receive non‐operative management according to the NCCN guidelines. Moreover, this study will demonstrate the possible benefit of TNT with respect to the “watch‐and‐wait” protocol.^34^

Even though short‐term outcomes such as pCR, nodal downstaging, and rate of R0 resection improve, data on long‐term outcomes are limited. There are many ongoing studies in which specific design, optimal sequences, types, durations of chemotherapy, ideal timings, and dosages of RT are not defined. In CAO/ARO/AIO‐12 (NCT02363374), group A (induction chemotherapy before CRT) was compared with group B (consolidation chemotherapy after CRT) to investigate the safety and efficacy of TNT sequences.^35^ Group B (CRT followed by consolidated chemotherapy) resulted in better compliance with CRT but worse compliance with chemotherapy than group A (induction chemotherapy). A better pCR rate was seen in group B. To adopt the TNT protocol as the primary treatment modality, well‐designed randomized controlled studies including molecular biomarkers will be necessary to determine the optimal treatment strategies according to patient characteristics. Nevertheless, TNT may be the preferred option in patients with LARC who have high risk factors for poor prognosis.

## 4 CONCLUSION

For the past decade, preoperative LCRT for LARC has been the standard treatment protocol for improving oncologic outcomes. With the accumulation of treatment experience, we identified groups of patients who did not show good tumor responses, with disease progression and early systemic failure as well. These findings suggest that one treatment plan cannot achieve all the oncological outcomes we desire.

Four robust consensus molecular subtypes (CMSs) were identified: CMS1, enriched for inflammatory/immune genes; CMS2, canonical; CMS3, metabolic; and CMS4, mesenchymal.^36^ CMS groups not only reflect cancer cell phenotypes but also microenvironmental features present in tumor tissue samples. Stage‐independent prognostic values and significant associations with multiple clinical and biological features were demonstrated. With respect to the chemorefractory setting, CMS2 (50%) followed by CMS4 (30%) were predominant in the CORRECT trial.^37^ The relatively consistent subtype distribution in first‐line trials may explain the enrichment of CMS2 and CMS4 over CMS3 and CMS1 after relapse in chemorefractory settings. Despite the potential clinical utility for outcome prediction or immune‐targeted therapy development, their clinical implementation is challenging. Prospective molecularly stratified clinical trials should be encouraged.^38^ The rapid evolution of knowledge in cancer biology should be integrated at the individual level.^39^

Advances in technologies such as imaging and molecular typing, combined with updated TNT protocols, may result in better oncological outcomes than current treatment strategies based on individual risk factors.

## DISCLOSURE

Conflict of Interest: The authors declare no conflicts of interest for this article.
